# Design of a Recessed Honeycomb Structure with a Nested Star Configuration and Study of Its Static Mechanical Properties

**DOI:** 10.3390/ma19112296

**Published:** 2026-05-28

**Authors:** Xinlin Wang, Guiwei Liu, Lei Lei, Weihang Peng

**Affiliations:** School of Mechanical Engineering, Dalian Jiaotong University, Dalian 116028, China; wxl_me@djtu.edu.cn (X.W.); lliuguiwei@163.com (G.L.); 18333136477@163.com (W.P.)

**Keywords:** negative Poisson’s ratio, CRNSC honeycomb structure, mechanical properties

## Abstract

Negative Poisson’s ratio materials show great potential in aerospace, automotive engineering, and military protection owing to their unique deformation behavior and superior mechanical properties. Nevertheless, current negative Poisson’s ratio honeycomb structures suffer from an inherent conflict between stiffness and energy absorption, along with poorly understood mechanical regulation mechanisms in complex three-dimensional nested configurations. To address these issues, this paper proposes a novel Cross Re-entrant Hexagon Nested Star-shaped Cell (CRNSC). Through theoretical derivation, finite element simulation, and quasi-static compression experiments, the mechanical properties and energy absorption characteristics of the structure are systematically investigated. A geometric characterization system based on length, angle, and thickness parameters is established. The results show that the cell wall thickness significantly increases the relative density, while the angle *θ* between the inner inclined strut and the horizontal line induces polarity reversal of the Poisson’s ratio. The outer inclined strut angle *α* and the inner angle *θ* exhibit monotonic or nonlinear regulatory effects on the equivalent Poisson’s ratio and the effective Young’s modulus, respectively. The optimal load-bearing configuration (*α* = 65°, *θ* = 35°) achieves a peak stress of 1.01 MPa, and the optimal deformation configuration (*α* = 55°, *θ* = 25°) reaches an ultimate strain of 4%. Theoretical, simulated, and experimental results are in good agreement with errors below 7%, validating the model’s effectiveness.

## 1. Introduction

The rapid advancement of modern industry has imposed increasingly stringent performance requirements on structural materials used in sectors such as aerospace, automotive manufacturing, naval engineering, and military defense. Conventional materials are progressively demonstrating inherent limitations in satisfying the combined requirements of lightweight construction, high strength, and enhanced energy absorption. This situation underscores an urgent necessity for the innovation of novel structural materials capable of transcending current performance constraints [1]. Metamaterials, a concept that emerged in the early 21st century, are characterized as artificially engineered materials or structures exhibiting unconventional physical properties [2,3]. In this context, negative Poisson’s ratio materials, owing to their unique deformation behavior and excellent mechanical properties, have become a prominent research focus within the field of metamaterials [4,5]. Also known as auxetic materials, negative Poisson’s ratio materials are characterized by lateral expansion under axial tension and lateral contraction under axial compression—a deformation mechanism that is diametrically opposite to the conventional Poisson’s effect observed in ordinary materials [6,7].

In 1987, Lakes successfully fabricated an artificial negative Poisson’s ratio foam material with a Poisson’s ratio of −0.7 using a thermal forming process on polyurethane foam [8]. Since then, Evans et al. [9] formally named the materials and structures with negative Poisson ‘s ratio effect as ‘ Auxetics ‘, marking the formation of negative Poisson ‘s ratio materials as an independent research field. When subjected to impact loads, the distinctive lateral contraction characteristic of negative Poisson’s ratio materials causes the material to converge toward the impacted region, resulting in an instantaneous increase in local density and thereby effectively enhancing impact resistance [10,11,12]. This property holds substantial application value in fields such as protective equipment and cushioning devices [13]. Furthermore, negative Poisson’s ratio materials exhibit functional properties including curved isotropy and variable permeability, presenting broad application prospects in areas such as biomedicine and smart sensors [14,15,16].

Honeycomb structures have been widely adopted in engineering applications owing to their lightweight nature, high specific strength, high specific stiffness, and excellent energy absorption capabilities [17,18]. Conventional hexagonal honeycomb structures exhibit a positive Poisson’s ratio effect under axial tension or compression. In 1982, Gibson et al. [19] first proposed the re-entrant hexagonal honeycomb structure. By indenting some edges of the conventional honeycomb structure inward, they achieved a negative Poisson’s ratio effect. Since then, scholars have conducted extensive research on negative Poisson’s ratio materials and structures [20,21,22,23], obtaining a series of novel two-dimensional honeycomb structures with negative Poisson’s ratio properties, including star-shaped, double-arrow, chiral, and rotating rigid-body structures [24,25,26]. These structures achieve the negative Poisson’s ratio effect through different deformation mechanisms, providing a rich design space for tailoring material properties.

Advances in 3D printing technology have provided robust support for the fabrication of complex negative Poisson’s ratio structures. Compared with traditional subtractive manufacturing methods, 3D printing offers distinct advantages, including high design flexibility, high material utilization, and short production cycles, making it particularly suitable for manufacturing negative Poisson’s ratio honeycombs with intricate internal architectures. Stereolithography (SLA) and digital light processing (DLP) 3D printing technologies are widely employed in the fabrication of negative Poisson’s ratio structures due to their high manufacturing precision. These technologies utilize ultraviolet light to selectively cure liquid photopolymer resins, enabling the monolithic fabrication of complex structures with micron-level precision.

Wang et al. [27] proposed a three-dimensional re-entrant negative Poisson’s ratio structure based on two-dimensional re-entrant hexagonal elements. Using beam theory, they derived theoretical expressions for the equivalent Young’s modulus and Poisson’s ratio, later validated by finite element simulations and compression tests. Yang et al. [28] systematically investigated three-dimensional star-shaped negative Poisson’s ratio structures and found that the design angle significantly influences impact resistance. Wang et al. [29] designed a re-entrant star-shaped three-dimensional negative Poisson’s ratio structure and examined its in-plane stiffness and energy absorption under low-, medium-, and high-speed impact conditions, showing substantially improved performance over conventional star-shaped honeycomb structures. Fu et al. [30] developed a three-dimensional chiral cubic lattice structure by spatially combining two-dimensional chiral honeycomb configurations. Theoretical analysis reveals that this structure exhibits an isotropic negative Poisson’s ratio effect, with a Poisson’s ratio reaching −1. Ebrahimi et al. [31] proposed a novel three-dimensional anti-chiral honeycomb metamaterial that achieves a negative Poisson’s ratio effect in three-dimensional space by connecting circular cells with anti-chiral topologies via inclined load-bearing struts.

Structural coupling strategies, which involve combining re-entrant structures with other negative Poisson’s ratio (NPR) structures, have emerged as a research hotspot in recent years. Researchers have found that integrating two existing structural elements to create a novel configuration—while preserving the negative Poisson’s ratio characteristics—represents an effective approach for designing NPR structures. Li et al. [32] proposed a novel NPR unit cell formed by nesting a square structure within a star-shaped structure and verified its NPR performance through micromechanical experiments and numerical simulations. Jin et al. [33] introduced a Re-entrant Hexagon Structure Nested Star-shaped Cell (RNSC) structure, consisting of a re-entrant hexagonal structure nested within a star-shaped structure. Through theoretical derivations, they obtained analytical expressions for its equivalent Young’s modulus and Poisson’s ratio. Their findings indicate that this structure overcomes the inherent limitation of re-entrant structures—the dependence of the negative Poisson’s ratio on the concavity angle—while effectively enhancing the Young’s modulus. Wei et al. [34] combined re-entrant hexagons with anti-chiral honeycombs to design a re-entrant anti-chiral honeycomb structure. The study confirmed that the novel structure exhibits superior energy absorption characteristics and a more pronounced negative Poisson’s ratio effect. Zhang et al. [35] proposed a hybrid star-re-entrant honeycomb structure, which demonstrates stable deformation patterns and excellent energy absorption performance under low-, medium-, and high-speed impact conditions.

Despite considerable advancements in the investigation of negative Poisson’s ratio honeycomb structures, numerous scientific and technical challenges remain unresolved. Firstly, existing negative Poisson’s ratio designs typically exhibit an intrinsic compromise between stiffness and energy absorption capacity; the high porosity characteristic of these structures often results in insufficient stiffness, thereby constraining their applicability in load-bearing contexts. Secondly, the majority of studies have concentrated on two-dimensional configurations or relatively simple three-dimensional forms, leaving the mechanical regulation mechanisms of complex three-dimensional nested architectures insufficiently understood. Thirdly, the precise quantitative relationship between structural geometric parameters and mechanical properties necessitates further clarification to facilitate accurate tailoring of material performance. Additionally, conventional re-entrant structures are susceptible to stress concentration and deformation instability under loading conditions, which undermines their load-bearing stability and diminishes their energy absorption efficiency.

To address these challenges, this paper proposes a novel Cross Re-entrant Hexagon Structure Nested Star-Shaped Cell (CRNSC). This three-dimensional negative Poisson’s ratio structure is constructed by embedding a two-dimensional star-shaped cell (SSC) within a re-entrant hexagonal cell (RHC) and subsequently intersecting them in a cross pattern. The proposed design aims to achieve synergistic optimization of the negative Poisson’s ratio effect and structural stiffness, offering new insights for the development of high-performance negative Poisson’s ratio materials.

## 2. Design of New Structures with Negative Poisson’s Ratio

### 2.1. Structural Design

**Figure 1:**
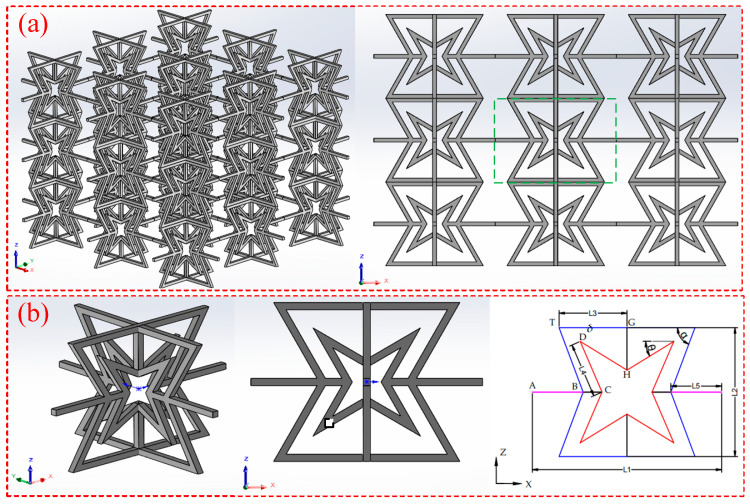
Novel cross-shaped negative Poisson’s ratio structure: (**a**) Schematic diagram of the periodic arrangement of the unit cell in three orthogonal directions; (**b**) Schematic diagram of the novel cross-shaped negative Poisson’s ratio unit cell structure.

As shown in Figure 1b, the novel CRNSC structure designed in this paper is a three-dimensional structure formed by embedding the SSC into the RHC and then intersecting them in a cross pattern. As shown in Figure 1a, the unit cell is assumed to be a representative volume element (RVE) of a triply periodic structure, characterized by 8 geometric parameters: five length parameters (L_1_, L_2_, L_3_, L_4_, L_5_), two angle parameters—α (the angle between the outer diagonal members of the unit cell) and θ (the angle between the inner diagonal members and the horizontal line)—and a thickness parameter δ. When subjected to loads in the x- or z-direction, the CRNSC exhibits negative Poisson’s ratio deformation. All structural members of the CRNSC unit cell have the same square cross-section. The side length of this square is denoted by δ (thickness parameter). As this study focuses on the effective Young’s modulus and equivalent Poisson’s ratio of the CRNSC within the elastic deformation range, appropriate forces are applied to ensure that the structural deformation remains elastic.

### 2.2. Derivation of Relative Density

The relative density of the CRNSC negative Poisson’s ratio structure is defined as the ratio of the equivalent density of the CRNSC unit cell to the density of the base material, expressed as follows:

(1) $$\rho_{rel}=\frac{\rho_{e}}{\rho_{m}}$$

where $\rho_{rel}$ is the relative density of the CRNSC, $\rho_{e}$ is the equivalent density of the CRNSC, and $\rho_{m}$ is the density of the solid base material.

The equivalent density of the CRNSC unit cell is defined as the actual volume of the constituent material within the unit cell divided by the total volume occupied by the unit cell, multiplied by the density of the material.

(2) $$\rho_{e}=\frac{\rho_{m}}{V_{1}}V_{2}$$

where $V_{1}$ and $V_{2}$ are the volumes occupied by the CRNSC unit cell and the solid material, respectively.

The relationship among the geometric parameters of the CRNSC unit cell is given by Equation (3):

(3) $$\left\{\begin{array}{c} L_{1}=2L_{5}+2L_{3}-2L_{3}cos\alpha \\ L_{2}=2L_{3}sin\alpha \\ L_{GH}=L_{3}sin\alpha +L_{4}sin\theta -L_{4}cos\theta \\ L_{BC}={L_{3}-L}_{3}cos\alpha +L_{4}sin\theta -L_{4}cos\theta \end{array}\right.$$

The volumes $V_{1}$ and $V_{2}$ are calculated as follows:

(4) $$\left\{\begin{array}{c} V_{1}=L_{1}^{2}L_{2}\\ V_{2}=2\left(8L_{3}+8L_{4}+2L_{5}+2L_{GH}+2L_{BC}\right)\delta^{2} \end{array}\right.$$

Substituting Equation (2) through (4) into Equation (1) yields:

(5) $$\rho_{rel}=\frac{\left[5L_{3}+4L_{4}+L_{5}+L_{3}sin\alpha -L_{3}cos\alpha +2(L_{4}sin\theta -L_{4}cos\theta )\right]\delta^{2}}{2{\left(L_{5}+L_{3}-L_{3}cos\alpha \right)}^{2}(L_{3}sin\alpha )}$$

### 2.3. Derivation of the Effective Young’s Modulus and Poisson’s Ratio

The deformation and displacement of the CRNSC are difficult to determine from the overall structure alone. Assuming that the external forces acting on the two intersecting parts are identical and uniformly distributed, the CRNSC unit cell exhibits geometric closed symmetry and statically indeterminate characteristics. The structure is symmetric about its center point (point symmetry), and the load distribution also satisfies this symmetry condition.

The overall stress analysis and load-strain calculation of the CRNSC are simplified to the stress–strain analysis of a 1/8 unit cell. By setting the boundaries of the 1/8 model as symmetry planes (with displacement or force constraints), the number of unknowns is significantly reduced. This transforms the complex statically indeterminate problem into a tractable boundary condition problem, allowing the key deformation parameters to be determined using only a few equations.

**Figure 2:**
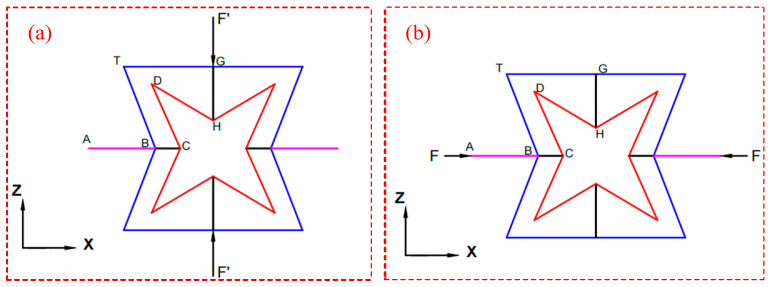
CRNSC Virtual Unit Load Distribution: (**a**) the *z*-axis direction; (**b**) the *x*-axis direction. *F* and *F*′ represent the pressures acting on a single two-dimensional planar unit cell of the CRNSC in the x-direction and z-direction, respectively. The uppercase letters A, B, C, D, H, G, and T denote the nodal points of the unit cell structure.

The analysis of the effective Young’s modulus and Poi sson’s ratio is based on Castigliano’s second theorem and the virtual unit load method. As shown in Figure 2, forces *F* and *F*′ are used to calculate the compressive loads in the x- and z-directions, respectively, for a single two-dimensional unit cell of the CRNSC structure. The horizontal compressive load F in the x-direction is shared by the RHC and the SSC; it is assumed that the RHC bears a compressive load of *F*_1_ and the SSC bears a compressive load of *F*_2_.

The sum of *F*_1_ and *F*_2_ equals the total horizontal load F acting in the x-direction of the unit cell, as shown in Equation (6).

(6) $$F=F_{1}+F_{\begin{array}{c} 2 \end{array}}$$

where *F* is the compressive load in the x-direction, and $F_{1}$ and $F_{2}$ are the compressive loads in the x-direction borne by the RHC and SSC, respectively.

The deformation of the CRNSC is primarily governed by bending strain energy (e.g., bending deformation of a member under load), while compressive and shear strain energies are relatively small and can be neglected. Therefore, only bending strain energy is considered when calculating nodal displacements. Since compressive strain energy is neglected, the members do not undergo axial expansion or contraction; consequently, the axial displacements at both ends of each member are equal. That is, the horizontal displacements at points B and C are equal, and the vertical displacements at points G and H are equal, as shown in Equation (7).

(7) $$\left\{\begin{array}{c} {∆}_{B}^{x}={∆}_{C}^{x}\\ {∆}_{H}^{Z}={∆}_{G}^{Z} \end{array}\right.$$

where ${∆}_{B}^{x}$ and ${∆}_{C}^{x}$ are the displacements of points B and C in the x-direction, respectively, and ${∆}_{H}^{Z}$ and ${∆}_{G}^{Z}$ are the displacements of points H and G in the z-direction, respectively.

**Figure 3:**
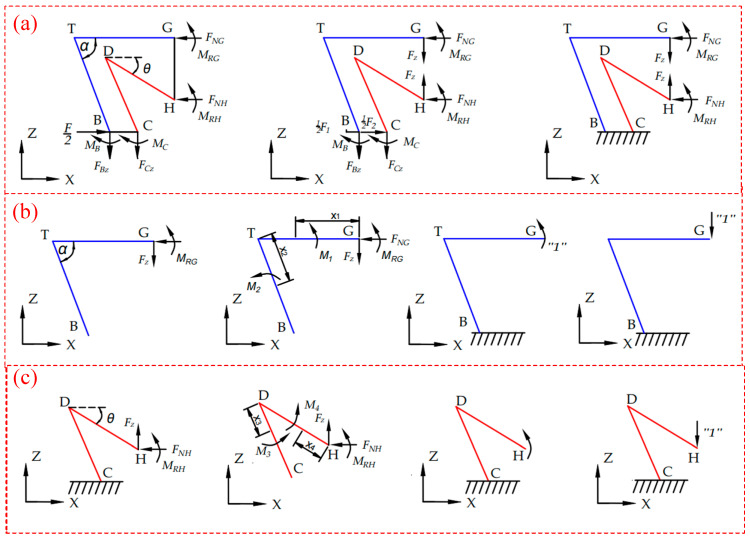
CRNSC x-Direction Load Analysis: (**a**) CRNSC load analysis; (**b**) RHC load analysis; (**c**) SSC load analysis.

The closed 1/8 CRNSC structure can be disassembled and simplified into two independent statically indeterminate structures, as shown in Figure 3a. There are 11 unknown internal forces on the symmetry plane: four moments $M_{B},\;M_{C},\;M_{RG},{\;M}_{RH}$ and seven forces $F_{1},\;F_{2},\;F_{Z},\;F_{BZ},\;F_{CZ},\;{F_{NG},\;F}_{NH}$. However, since only six equilibrium equations are available, the two two-dimensional statically indeterminate structures possess five indeterminate unknowns. Therefore, in addition to Equation (6), four constraint equations are required to determine all unknowns. This paper employs Castigliano’s Second Theorem and the virtual unit load method to solve this statically indeterminate problem.

Since sections H and G are symmetry planes of the structure, their rotation angles must be zero. In the following, the relationship between the section rotation angles will be derived using the energy method, which requires solving for the unknowns $M_{RG}\;and{\;M}_{RH}$. Applying Saint-Venant’s principle, sections B and C can be treated as fixed constraints, allowing the RHC and SSC to be analyzed separately.

As shown in Figure 3b, the analysis of the RHC is performed by fixing section B and using the rotation angle of section G as the deformation compatibility condition. The analysis indicates that this structure is a first-order statically indeterminate structure, and its canonical equation is as follows:

(8) $$\delta_{11}\cdot X_{1}+{∆}_{1F}\;=0$$

where $\delta_{11}$ is the rotation angle of section G when a virtual unit load is applied; ${∆}_{1F}$ is the rotation angle of section G when the original load is applied; and $X_{1}$ is the redundant constraint force.

To determine the rotation angle of section G when loads $F_{Z}$ and $F_{NG}$ are applied, Mohr’s theorem is used to integrate over members GT and TB, respectively.

(9) $$\begin{array}{c} {∆}_{1F}=\int_{0}^{L_{3}}\frac{M_{1}\left(x_{1}\right)}{EI}\frac{\partial M_{1}\left(x_{1}\right)}{\partial F_{Z}}dx_{1}+\int_{0}^{L_{3}}\frac{M_{2}\left(x_{2}\right)}{EI}\frac{\partial M_{1}\left(x_{2}\right)}{\partial F_{1}}+\frac{M_{2}\left(x_{2}\right)}{EI}\frac{\partial M_{2}\left(x_{2}\right)}{\partial F_{Z}}dx_{2} \end{array}\;=\frac{1}{EI}\left(\int_{0}^{L_{3}}-F_{Z}x_{1}^{2}dx_{1}+\int_{0}^{L_{3}}\frac{F_{1}}{4}x_{2}^{2}sin^{2}\alpha -F_{Z}x_{2}^{2}cos^{2}\alpha dx_{2}\right)\;=\frac{F_{1}L_{3}^{3}sin^{2}\alpha -4F_{Z}L_{3}^{3}cos^{2}\alpha -{4F_{Z}L}_{3}^{3}}{12EI}$$

where $x_{1}$ and $x_{2}$ are the displacement variables along the cross-sections of members GT and TB, respectively; $M_{1}$ and $M_{2}$ are the bending moments at sections $x_{1}$ and $x_{2}$, respectively; E is the Young’s modulus of the material; and I is the second moment of area of the member. In Figure 3b, when only a unit bending moment is applied, the expression for $\delta_{11}$ is as follows:

(10) $$\delta_{11}=\frac{L_{3}^{3}sin\alpha }{3EI}$$

Substituting Equations (9) and (10) into Equation (8), the expression for $X_{1}$ is obtained as follows:

(11) $$X_{1}=-\frac{{∆}_{1F}}{\delta_{11}}=\frac{1+cos^{2}\alpha }{sin\alpha }F_{Z}-sin\alpha F_{1}$$

(12) $$M_{RG}=L_{3}\cdot X_{1}=L_{3}\left(\frac{1+cos^{2}\alpha }{sin\alpha }F_{Z}-sin\alpha F_{1}\right)$$

When loads $F_{N}$ and $F_{z}$, along with bending moment MRG, are applied simultaneously, Castigliano’s second theorem can be used to derive the displacement ${∆}_{B}^{x}$ of the RHC in the x-direction and the displacement ${∆}_{G}^{z}$ in the z-direction. Similarly, analysis of the SSC (Figure 3c) yields its displacement ${∆}_{C}^{x}$ in the x-direction and ${∆}_{H}^{z}$ in the z-direction.

(13) $$\left\{\begin{array}{c} {∆}_{B}^{x}=\xi_{11}F_{1}+\xi_{12}F_{Z}\\ {∆}_{C}^{x}=\xi_{13}F_{2}+\xi_{14}F_{Z}\\ {∆}_{G}^{z}=\xi_{21}F_{1}+\xi_{22}F_{Z}\\ {∆}_{H}^{z}=\xi_{23}F_{2}+\xi_{24}F_{Z} \end{array}\right.$$

(14) $$\left\{\begin{array}{c} \xi_{11}=\frac{5L_{1}^{3}sin^{2}\alpha }{24EI}\xi_{12}=\frac{L_{1}^{3}sin\alpha (5cos\alpha -3)}{12EI}\\ \xi_{13}=-\frac{L_{4}^{3}(3sin2\theta -5)}{24EI}\xi_{14}=-\frac{L_{4}^{3}(5sin2\theta -3)}{12EI}\\ \xi_{21}=\frac{6L_{1}^{3}sin^{2}\alpha -5L_{1}^{3}sin2\alpha }{48EI}\xi_{22}=-\frac{5L_{1}^{3}cos^{2}\alpha +5L_{1}^{3}-6L_{1}^{3}cos\alpha }{12EI}\\ \xi_{23}=-\frac{L_{4}^{3}(5sin2\theta -3)}{24EI}\xi_{24}=-\frac{L_{4}^{3}(3sin2\theta -5)}{12EI} \end{array}\right.$$

(15) $$\left\{\begin{array}{c} F_{1}=K_{1}F\\ F_{2}=K_{2}F\\ F_{Z}=K_{3}F\\ K_{1}=\frac{\frac{\xi_{13}}{\xi_{14}-\xi_{12}}-\frac{\xi_{23}}{\xi_{24}-\xi_{22}}}{\frac{\xi_{11}+\xi_{13}}{\xi_{14}-\xi_{12}}-\frac{\xi_{21}{+\xi }_{23}}{\xi_{24}-\xi_{22}}}\\ =\frac{\frac{L_{4}^{3}(5sin2\theta -3)}{\omega_{1}}+\frac{L_{4}^{3}(3sin2\theta -5)}{\omega_{2}}}{\frac{L_{4}^{3}\left(5sin2\theta -3\right)+\omega_{3}}{\omega_{1}}+\frac{L_{4}^{3}\left(3sin2\theta -5\right)-2\omega_{4}}{\omega_{2}}}\\ K_{2}=1-K_{1}\\ K_{3}=\frac{\xi_{11}+\xi_{13}}{\xi_{14}-\xi_{12}}K_{1}-\frac{\xi_{13}}{\xi_{14}-\xi_{12}}\\ =\frac{L_{4}^{3}\left(3sin2\theta -5\right)-2\omega_{4}}{24\omega_{2}}K_{1}\\ \omega_{1}=\frac{5L_{3}^{3}+5L_{3}^{3}cos^{2}\alpha -6L_{3}^{3}cos\alpha -L_{4}^{3}\left(3sin2\theta -5\right)}{12}\\ \omega_{2}=\frac{L_{4}^{3}\left(5sin2\theta -3\right)-\omega_{3}}{12}\\ \omega_{3}=\frac{5L_{3}^{3}sin2\alpha -6L_{3}^{3}sin\alpha }{2}\\ \omega_{4}=\frac{5L_{3}^{3}sin2\alpha }{2} \end{array}\right.$$

The effective Young’s modulus is calculated based on the average stress and average strain theorem, as follows:

(16) $$\left\{\begin{array}{c} E=\frac{\sigma }{\varepsilon }\\ \sigma =\frac{F}{A^{*}}\\ \varepsilon =\frac{∆l}{L^{*}} \end{array}\right.$$

From Figure 1, the equivalent length and equivalent cross-sectional area of the CRNSC in the x-direction are given as follows:

(17) $$\left\{\begin{array}{c} L_{x}^{*}=2L_{5}+2L_{3}-2L_{3}cos\alpha \\ A_{x}^{*}=\delta \left(2L_{3}sin\alpha \right) \end{array}\right.$$

Poisson’s ratio is defined as the negative ratio of the absolute transverse strain to the absolute axial strain, characterizing the transverse deformation behavior of a material under uniaxial tension or compression:

(18) $$v_{yx}=-\frac{\varepsilon_{x}}{\varepsilon_{y}}$$

To obtain the deformations at sections B and C, substitute Equations (15) and (17) into Equations (13) and (14) to obtain the forces $F_{1}$, $F_{2}$, and $F_{z}$. Substituting the above results together with Equation (17) into Equations (16) and (18) yields the effective Young’s modulus $E_{zx}^{eq}$ and the equivalent Poisson’s ratio $v_{zx}^{eq}$ of the CRNSC under an x-direction load, as follows:

(19) $$\left\{\begin{array}{c} E_{zx}^{eq}=\frac{E\delta^{3}(L_{5}+L_{3}-L_{3}cos\alpha )}{L_{4}^{3}\left(L_{3}sin\alpha \right)\left[\left(5sin2\theta -3\right)\eta_{1}+(3sin2\theta -5)\eta_{2}\right]}\\ v_{zx}^{eq}=\frac{(L_{5}+L_{3}-L_{3}cos\alpha )\left[\left(5sin2\theta -3\right)\eta_{1}+2(3sin2\theta -5)\eta_{3}\right]}{\left(L_{3}sin\alpha \right)\left[2\left(5sin2\theta -3\right)\eta_{1}+(3sin2\theta -5)\eta_{3}\right]} \end{array}\right.$$

(20) $$\left\{\begin{array}{c} \begin{array}{c} \eta_{1}=\frac{L_{4}^{3}(3sin2\theta -5)}{24\omega_{2}}-\frac{\begin{array}{c} L_{4}^{3}[\omega_{2}\left(5sin2\theta -3\right)+\omega_{1}(3sin2\theta -5)](3L_{4}^{3}sin2\theta -5L_{4}^{3}-2\omega_{4}) \end{array}}{24\omega_{2}\left[12\omega_{2}^{2}+\omega_{1}(3L_{4}^{3}sin2\theta -5L_{4}^{3}-2\omega_{4})\right]}\\ \eta_{2}=\frac{L_{4}^{3}[\omega_{2}\left(5sin2\theta -3\right)+\omega_{1}(3sin2\theta -5)]}{24\omega_{2}+2\omega_{1}(3L_{4}^{3}sin2\theta -5L_{4}^{3}-2\omega_{4})}-\frac{1}{2}\\ \eta_{3}=\frac{L_{4}^{3}[\omega_{2}\left(5sin2\theta -3\right)+\omega_{1}(3sin2\theta -5)]}{\omega_{2}\left[12\omega_{2}^{2}+\omega_{1}(3L_{4}^{3}sin2\theta -5L_{4}^{3}-2\omega_{4})\right]}-1 \end{array} \end{array}\right.$$

**Figure 4:**
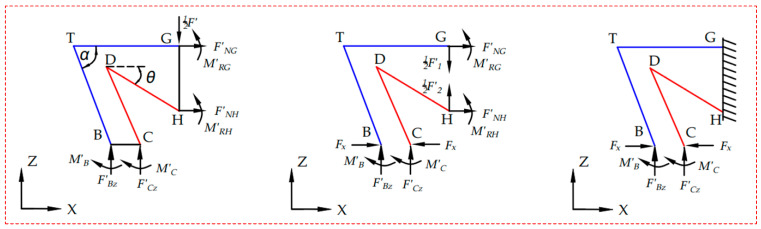
CRNSC z-Direction Load Analysis.

Figure 4 presents the analysis and calculation of the compressive load on the CRNSC in the z-direction. The derivation process is similar to that for the x-direction.

(21) $$\left\{\begin{array}{c} {∆}_{B}^{x}={∆}_{C}^{x}\\ {∆}_{H}^{Z}={∆}_{G}^{Z} \end{array}\right.$$

(22) $$\left\{\begin{array}{c} {∆}_{B}^{x}={\xi^{\prime }}_{11}{F^{\prime }}_{1}+{\xi^{\prime }}_{12}F_{X}\\ {∆}_{C}^{x}={\xi^{\prime }}_{13}F_{2}+{\xi^{\prime }}_{14}F_{X}\\ {∆}_{G}^{y}={\xi^{\prime }}_{21}{F^{\prime }}_{1}+{\xi^{\prime }}_{22}F_{X}\\ {∆}_{H}^{y}={\xi^{\prime }}_{23}{F^{\prime }}_{2}+{\xi^{\prime }}_{24}F_{X} \end{array}\right.$$

(23) $$\left\{\begin{array}{c} {\xi^{\prime }}_{11}=\frac{5L_{1}^{3}cos^{2}\alpha +5L_{1}^{3}-6L_{1}^{3}cos\alpha }{24EI}{\xi^{\prime }}_{12}=\frac{5L_{1}^{3}sin2\alpha -6L_{1}^{3}sin^{2}\alpha }{24EI}\\ {\xi^{\prime }}_{13}=-\frac{L_{4}^{3}(3sin2\theta -5)}{24EI}{\xi^{\prime }}_{14}=-\frac{L_{4}^{3}(5sin2\theta -3)}{12EI}\\ {\xi^{\prime }}_{21}=\frac{6L_{1}^{3}sin^{2}\alpha -5L_{1}^{3}sin2\alpha }{48EI}{\xi^{\prime }}_{22}=\frac{5L_{1}^{3}sin^{2}\alpha }{12EI}\\ {\xi^{\prime }}_{23}=-\frac{L_{4}^{3}(5sin2\theta -3)}{24EI}{\xi^{\prime }}_{24}=-\frac{L_{4}^{3}(3sin2\theta -5)}{12EI} \end{array}\right.$$

(24) $$\left\{\begin{array}{c} {F^{\prime }}_{1}={K^{\prime }}_{1}F^{\prime }\\ {F^{\prime }}_{2}={K^{\prime }}_{2}F^{\prime }\\ F_{x}={K^{\prime }}_{3}F^{\prime }\\ {K^{\prime }}_{1}=\frac{\frac{{\xi^{\prime }}_{13}}{{\xi^{\prime }}_{14}-{\xi^{\prime }}_{12}}-\frac{{\xi^{\prime }}_{23}}{{\xi^{\prime }}_{24}-{\xi^{\prime }}_{22}}}{\frac{{\xi^{\prime }}_{11}+{\xi^{\prime }}_{13}}{{\xi^{\prime }}_{14}-{\xi^{\prime }}_{12}}-\frac{{\xi^{\prime }}_{21}{+\xi^{\prime }}_{23}}{{\xi^{\prime }}_{24}-{\xi^{\prime }}_{22}}}\\ =\frac{\frac{L_{4}^{3}(5sin2\theta -3)}{L_{4}^{3}(3sin2\theta -5)-2\omega_{4}}-\frac{L_{4}^{3}(3sin2\theta -5)}{12\omega_{2}}}{\frac{\omega_{1}}{\omega_{2}}+\frac{12\omega_{2}}{L_{4}^{3}\left(3sin2\theta -5\right)-2\omega_{4}}}\\ {K^{\prime }}_{2}=1-{K^{\prime }}_{1}\\ {K^{\prime }}_{3}=\frac{{\xi^{\prime }}_{11}+{\xi^{\prime }}_{13}}{{\xi^{\prime }}_{14}-{\xi^{\prime }}_{12}}{K^{\prime }}_{1}-\frac{{\xi^{\prime }}_{13}}{{\xi^{\prime }}_{14}-{\xi^{\prime }}_{12}}\\ =-\frac{L_{4}^{3}\left(3sin2\theta -5\right)-2{4\omega }_{1}}{24\omega_{2}}{K^{\prime }}_{1}\\ \omega_{1}=\frac{5L_{3}^{3}+5L_{3}^{3}cos^{2}\alpha -6L_{3}^{3}cos\alpha -L_{4}^{3}\left(3sin2\theta -5\right)}{12}\\ \omega_{2}=\frac{L_{4}^{3}\left(5sin2\theta -3\right)-\omega_{3}}{12}\\ \omega_{3}=\frac{5L_{3}^{3}sin2\alpha -6L_{3}^{3}sin\alpha }{2}\\ \omega_{4}=\frac{5L_{3}^{3}sin2\alpha }{2} \end{array}\right.$$

The equivalent length and equivalent cross-sectional area of the CRNSC in the z-direction are as follows:

(25) $$\left\{\begin{array}{c} L_{z}^{*}=2L_{3}sin\alpha \\ A_{z}^{*}=\delta \left(2L_{5}+2L_{3}-2L_{3}cos\alpha \right) \end{array}\right.$$

Similarly, by substituting Equations (22)–(24) into Equation (21), the forces *F*_1_, *F*_2_ and *F_x_* can be obtained. Substituting these results together with Equation (25) into Equations (16) and (18) yields the effective Young’s modulus $E_{xz}^{eq}$ and the equivalent Poisson’s ratio $v_{xz}^{eq}$ of the CRNSC under a load in the z-direction, as follows:

(26) $$\left\{\begin{array}{c} E_{xz}^{eq}=\frac{E\delta^{3}(L_{3}sin\alpha )}{L_{4}^{3}\left(L_{5}+L_{3}-L_{3}cos\alpha \right)\left[\left(5sin2\theta -3\right){\eta^{\prime }}_{1}+(3sin2\theta -5){\eta^{\prime }}_{2}\right]}\\ v_{xz}^{eq}=\frac{(L_{3}sin\alpha )\left[\left(5sin2\theta -3\right){\eta^{\prime }}_{3}+2(3sin2\theta -5){\eta^{\prime }}_{1}\right]}{\left(L_{5}+L_{3}-L_{3}cos\alpha \right)\left[2\left(5sin2\theta -3\right){\eta^{\prime }}_{1}+(3sin2\theta -5){\eta^{\prime }}_{3}\right]} \end{array}\right.$$

(27) $$\left\{\begin{array}{c} {\eta^{\prime }}_{1}=\frac{L_{4}^{3}(3sin2\theta -5)}{24\omega_{2}}+\frac{\begin{array}{c} L_{4}^{3}\omega_{1}[12\omega_{2}\left(5sin2\theta -3\right)-\left(3sin2\theta -5\right)\left(3L_{4}^{3}sin2\theta -5L_{4}^{3}-2\omega_{4}\right)] \end{array}}{24\omega_{2}\left[12\omega_{2}^{2}+\omega_{1}(3L_{4}^{3}sin2\theta -5L_{4}^{3}-2\omega_{4})\right]}\\ {\eta^{\prime }}_{2}=\frac{L_{4}^{3}[12\omega_{2}\left(5sin2\theta -3\right)-(3sin2\theta -5)\left(3L_{4}^{3}sin2\theta -5L_{4}^{3}-2\omega_{4}\right)]}{24\omega_{2}\left[12\omega_{2}^{2}+\omega_{1}(3L_{4}^{3}sin2\theta -5L_{4}^{3}-2\omega_{4})\right]}-\frac{1}{2}\\ {\eta^{\prime }}_{3}=\frac{L_{4}^{3}[12\omega_{2}\left(5sin2\theta -3\right)+(3sin2\theta -5)]}{12\left[12\omega_{2}^{2}+\omega_{1}(3L_{4}^{3}sin2\theta -5L_{4}^{3}-2\omega_{4})\right]}-1 \end{array}\right.$$

### 2.4. Parameter Analysis

Based on the analytical expressions above, this section further analyzes the correlations between the geometric parameters of the CRNSC and its relative density, effective Young’s modulus, and equivalent Poisson’s ratio. A univariate parameter analysis method is adopted, in which only one parameter is varied at a time while the others are held constant. The independent parameters considered in the sensitivity analysis include: the angle *α* between the outer diagonal rods of the unit cell, the angle *θ* between the inner diagonal rods and the horizontal line, the unit cell thickness parameter *δ*, and the rod length parameters *L*_3_, *L*_4_, and *L*_5_. The fixed parameter set consists of *α* = 60°, *θ* = 30°, *δ* = 2 mm, *L*_3_ = 24 mm, *L*_4_ = 14 mm, and *L*_5_ = 20 mm. The variation range of each parameter is listed in Table 1.

#### 2.4.1. Relationship Between Geometric Parameters and Relative Density

**Figure 5:**
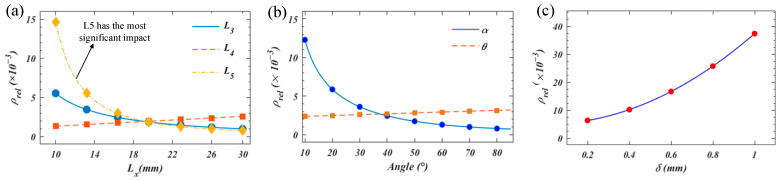
Relationship between geometric parameters and relative density. (**a**) relative density vs. rod length parameters; (**b**) relative density vs. angle parameters; (**c**) relative density vs. thickness parameter.

Figure 5 illustrates the relationship between various geometric parameters and the relative density $\rho_{\mathrm{rel}}$. As shown, all geometric parameters influence $\rho_{\mathrm{rel}}$ by altering the material volume fraction within the structure. Among the rod length parameters, increasing *L*_3_ and *L*_5_ causes $\rho_{\mathrm{rel}}$ to decrease significantly, with *L*_5_ having a more pronounced effect, while *L*_4_ has only a mild influence. This is because *L*_3_ and *L*_5_ directly determine the overall spatial extent of the structure; their increase makes the structure excessively “stretched” in the corresponding direction, substantially reducing the material volume fraction relative to the total geometric space. In contrast, *L*_4_, representing a local rod length, exerts a weaker regulatory effect on the overall material volume fraction. Regarding the angle parameters, an increase in *α* reduces $\rho_{\mathrm{rel}}$, whereas an increase in *θ* raises $\rho_{\mathrm{rel}}$. This occurs because *α* controls the opening degree of the outer oblique structure; the larger *α* is, the more “spread out” the structure becomes in space, resulting in a lower material volume fraction. Conversely, *θ* relates to the compactness of the internal star-shaped structure; the larger *θ* is, the more clustered the internal structure becomes, leading to a higher material volume fraction. The thickness parameter *δ* is positively correlated with $\rho_{\mathrm{rel}}$, as *δ* determines the material thickness; a larger *δ* increases the volume fraction of material filling the structure, and consequently, $\rho_{\mathrm{rel}}$ rises significantly.

#### 2.4.2. Relationship Between Geometric Parameters and the Equivalent Poisson’s Ratio

**Figure 6:**
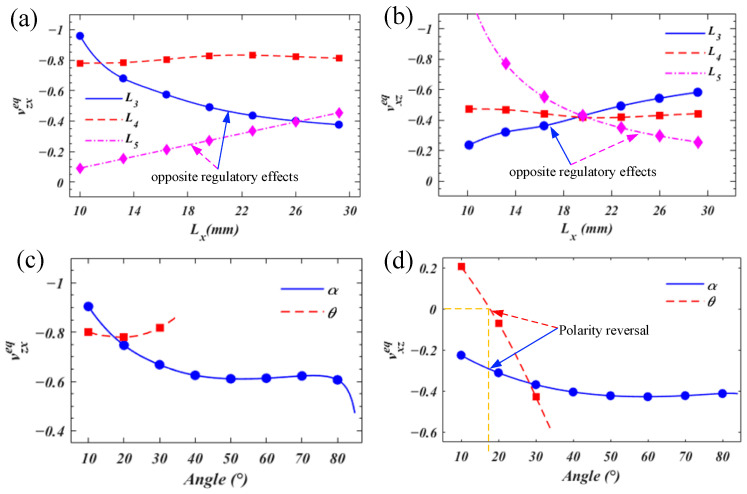
Effect of geometric parameters on equivalent Poisson’s ratio. (**a**) rod length parameters (x-direction); (**b**) rod length parameters (z-direction); (**c**) angle parameters (x-direction); (**d**) angle parameters (z-direction).

Figure 6 illustrates the relationship between geometric parameters and the equivalent Poisson’s ratio. The angle parameters *α* and *θ*, together with the rod length parameters *L*_3_, *L*_4_, and *L*_5_, exert distinct regulatory effects on the equivalent negative Poisson’s ratio in the x- and z-directions. This is because the geometric configuration of the auxetic structure directly governs deformation compatibility, stress transfer paths, and local deformation modes under loading, thereby fundamentally determining its equivalent mechanical properties.

The length parameters *L*_3_, *L*_4_, and *L*_5_ differentially regulate the deformation coupling efficiency in orthogonal directions by altering the dimensional ratios of functional segments, stiffness distribution, and deformation constraint boundaries, thus enabling directional decoupling control of the equivalent Poisson’s ratio. *L*_3_ determines the length of the outer load-bearing arm, regulating the overall degree of deformation constraint and the coupling efficiency between longitudinal and transverse deformations. As *L*_3_ increases, the negative magnitude of the equivalent Poisson’s ratio in the x-direction exhibits a significant monotonically decreasing trend, while that in the z-direction shows a synchronously increasing trend; *L*_3_ is therefore the key length parameter for achieving opposite regulation of the Poisson’s ratio in orthogonal directions. *L*_5_ exhibits a decoupling effect opposite to that of *L*_3_, as it directly alters the vertical dimension of the internal core deformation unit. As *L*_5_ increases, the negative magnitude of the equivalent Poisson’s ratio in the x-direction increases monotonically, whereas that in the z-direction decreases monotonically; the combination of *L*_5_ and *L*_3_ enables independent tuning of the Poisson’s ratio in orthogonal directions. *L*_4_ primarily determines the dimension of the central load-bearing segment. Across the entire parameter range, it exerts minimal influence on the equivalent Poisson’s ratios in both directions, serving mainly to maintain overall structural stiffness and ensure stable negative Poisson’s ratio characteristics; *L*_4_ thus acts as a benchmark parameter for structural performance stability.

The angle parameter *α* determines the inclination of the diagonal load-bearing members in the structural core, directly affecting the coupling ratio between deformations in the x- and z-directions under loading. It exerts a continuous, gradual, and nonlinear regulatory effect on the equivalent Poisson’s ratios in both orthogonal directions, enabling fine-tuning of the Poisson’s ratio over a wide range. The angle parameter *θ*, in contrast, controls the opening and closing of the sharp corners of the internal deformation units, thereby altering the degree of local deformation concentration and the primary stress transfer path. It can directly trigger a polarity reversal of the equivalent Poisson’s ratio in the z-direction as well as rapid changes in the x-direction, making it a highly sensitive parameter for precise customization of the Poisson’s ratio. By altering the fundamental deformation mechanism of the structure, these two parameters cause the equivalent Poisson’s ratio to exhibit distinctly different range-dependent characteristics.

#### 2.4.3. Relationship Between Geometric Parameters and Effective Young’s Modulus

**Figure 7:**
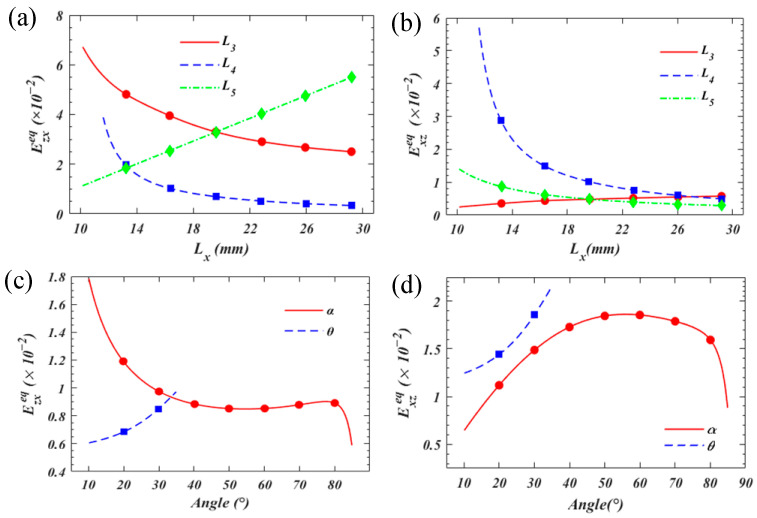
Effective Young’s modulus vs. geometric parameters: (**a**) x-direction vs. rod length; (**b**) z-direction vs. rod length; (**c**) x-direction vs. angle; (**d**) z-direction vs. angle.

Figure 7 illustrates the relationship between geometric parameters and the effective Young’s modulus. As shown in the figure, geometric parameters regulate the elastic response behavior of the structure by altering the force transmission path, the characteristics of the effective load-bearing cross-section, and the deformation mode. Within the elastic deformation range, the effective Young’s modulus of the structure is primarily governed by the relative density, which is directly determined by the geometric parameters, with porosity and pore morphology being the key geometric factors. In the CRNSC structure, *L*_4_, *α*, and *θ* have the most significant influence on the effective Young’s modulus, while the regulatory effects of *L*_3_ and *L*_5_ are relatively mild. The influence of each parameter on the effective Young’s modulus in the x- and z-directions exhibits distinct patterns:

*L*_4_ is a key parameter affecting the effective Young’s modulus, as it directly determines the “flexibility” of the star-shaped re-entrant region. A larger *L*_4_ significantly increases the elastic deformation space in this region, markedly weakening the overall load-bearing stiffness of the structure. Consequently, the effective Young’s modulus in both directions decreases sharply as *L*_4_ increases, exhibiting the most pronounced attenuation effect. *α* exerts a nonlinear regulatory effect on the effective Young’s modulus by directly controlling the force transmission efficiency and stiffness distribution of the sidewall structure. In the x-direction, as *α* increases, the load-bearing contribution of the sidewall structure to the overall load continuously decreases, making the structure more prone to bending deformation. Thus, the effective Young’s modulus in the x-direction shows a significant downward trend with increasing *α*, leveling off only in the mid-angle range. In the z-direction, as *α* increases, the effective Young’s modulus first rises and then falls, reaching a stiffness peak in the mid-angle range, while both the low-angle and high-angle ranges weaken the overall stiffness in the z-direction. *θ* exerts a positive regulatory effect on the effective Young’s modulus by influencing the inclination of the star-shaped arms and altering the distribution of component forces and deformation patterns during force transmission. As *θ* increases, the inclination angle of the star-shaped arms is optimized, the overall deformation resistance of the structure is enhanced, and load transfer efficiency is significantly improved. Therefore, the effective Young’s modulus in both directions exhibits a continuous upward trend with increasing *θ*, and its enhancing effect on z-direction stiffness is far stronger than that on x-direction stiffness, making *θ* the core angular parameter for regulating the structure’s z-direction stiffness.

## 3. Static Mechanical Properties of the CRNSC Unit Cell

### 3.1. Finite Element Modeling

This section focuses on analyzing the influence of the angle parameters *α* and *θ* on the equivalent mechanical properties of the CRNSC structure. The remaining geometric parameters were selected based on three key performance objectives: high relative density, a balanced negative Poisson’s ratio effect, and a moderate effective Young’s modulus. The values of α and *θ* were set to ±5° relative to the angle parameters of the positive RHC and positive SSC structures, and the specific geometric parameters are shown in Table 2.

**Figure 8:**
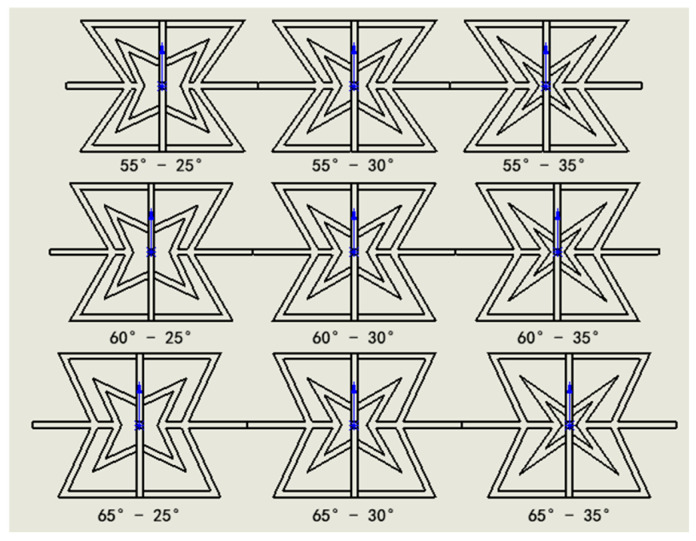
9-Group Angle-Parameter Orthogonal Simulation View.

The CRNSC unit cell structures for the orthogonal experiments with nine sets of angle parameters were modeled using SolidWorks 2022, and the corresponding engineering views of the simulation are shown in Figure 8.

Static structural analysis was performed using the Static Structural module in the Ansys Workbench (2021 R1) platform. The matrix material of the CRNSC structure is high-toughness white ABS resin, and its material properties are listed in Table 3.

**Figure 9:**
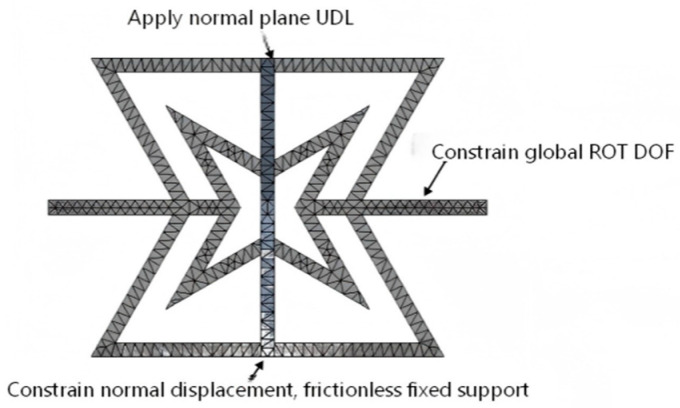
Schematic of Boundary and Load Conditions for Simulated Structure.

As illustrated in Figure 9, which shows the boundary and loading conditions of the simulated structure, the mesh for the CRNSC unit cell is strictly controlled to ensure that the Jacobian determinant is greater than 0.7 and the element aspect ratio is less than 5. The mesh size is set to 1 mm to balance computational accuracy and solution efficiency. Rotational degrees of freedom are constrained, while transverse deformation degrees of freedom are retained. During compression, only the normal displacement of the bottom surface is fixed, allowing free transverse deformation. The asymmetry observed in the simulation results arises directly from the asymmetric boundary conditions, where only the normal displacement of the bottom surface is constrained while lateral deformation is unrestricted. This setup does not enforce symmetric transverse deformation, allowing the structure to deform freely. The observed asymmetry is therefore a physical consequence of the boundary conditions rather than a modeling error, and it does not compromise the validity of the derived mechanical properties. This boundary condition setup yields realistic equivalent Poisson’s ratio results that fully align with the deformation compatibility conditions of the theoretically simplified 1/8 symmetric model, thereby avoiding over-constraint issues that could lead to overestimated stiffness and distorted negative Poisson’s ratio effects. The maximum load is F = 250 N, applied as a uniformly distributed planar load to ensure that the load distribution matches the theoretical assumptions. For large-deformation analysis, displacement-controlled loading is employed to improve solution convergence.

### 3.2. Equivalent Poisson’s Ratio Analysis

**Figure 10:**
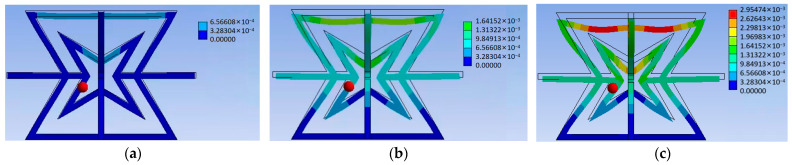
Schematic illustration of the deformation process of a unit cell structure: (**a**) Initial deformation; (**b**) Mid-deformation; (**c**) Final deformation.

Figure 10 shows the total deformation contour plot of the CRNSC structure under the parameter set *α* = 60° and *θ* = 30°. It can be observed that the structure exhibits a regular deformation evolution and a typical negative Poisson’s ratio mechanical response when subjected to normal loads. Based on the loading process, the deformation process can be divided into three stages:

(1) Linear elastic stage. Under low loads, the structure remains within the linear elastic range. Deformation is primarily driven by rigid-body rotation at the nodes of the re-entrant diagonal beams, accompanied only by minor bending deformation. The compressive load at the top is transmitted downward through the internal vertical center beam, inducing downward axial displacement. The lower end of the re-entrant diagonal beam is connected to the fixed support at the bottom, which fully constrains its vertical displacement. The displacement difference between the upper and lower ends forces the diagonal beam to rotate outward about the nodes as a rigid body, thereby driving the intermediate horizontal links to undergo synchronous lateral displacement on both sides, resulting in lateral expansion under axial compression. At this stage, the negative Poisson’s ratio effect begins to emerge, with deformation gradually decreasing from the top loading end toward the bottom fixed support. The overall deformation distribution follows the linear elastic transfer law.
(2) Nonlinear stage. As the load increases, the structure enters the moderate deformation stage. At this point, the deformation mode shifts toward coupling of rigid-body rotation and beam bending deformation; geometric nonlinear effects become pronounced, and structural stiffness gradually softens. The axial displacement of the central beam increases significantly, and the rotation amplitude of the re-entrant diagonal beams increases correspondingly. Meanwhile, the proportion of bending deformation rises rapidly, and the structure no longer undergoes purely rigid-body motion. This change causes the negative Poisson’s ratio effect to enter a stage of nonlinear growth, with the ratio of lateral expansion to axial compression continuing to increase. The high-deformation zone expands significantly from the center of the top crossbeam toward both sides, while the decay rate of the deformation gradient gradually slows; bending deformation has become a core contributor to the structural deformation.
(3) Strong nonlinear stage. When the load reaches a sufficiently high level, the structure enters the large-deformation stage. Large bending deformation of the re-entrant diagonal beams becomes dominant, accompanied by strong coupling between large rotation and axial tension-compression of the beams, driving the structure into a regime of strong geometric nonlinearity. Under the applied load, the re-entrant diagonal beams are nearly straightened, and their original re-entrant configuration is significantly altered. The ultimate outward opening of the diagonal beams drives the lateral expansion of the central horizontal link to its peak. Compared with the original undeformed profile, the typical negative Poisson’s ratio deformation characteristic—where the structure is “compressed vertically and expanded laterally”—is clearly visible, and the negative Poisson’s ratio effect is fully manifested at this stage. If loading continues, once the diagonal beams become fully straightened, the structure enters a stiffness-hardening phase. Subsequent deformation must overcome the axial stiffness of the beams, and the negative Poisson’s ratio effect gradually diminishes.

In summary, under the continuous action of normal loads, the re-entrant unit cell structure of the CRNSC undergoes cooperative deformation: the overall structure compresses along the normal direction of the applied load while simultaneously contracting and converging toward the central axis in the transverse direction. This deformation behavior fully conforms to the core mechanical definition of negative Poisson’s ratio structures, demonstrating excellent and stable negative Poisson’s ratio mechanical properties. It also validates the effectiveness of the deformation compatibility and load-bearing mechanical response of the CRNSC structure under this parameter configuration, as predicted by the theoretical analysis.

**Figure 11:**
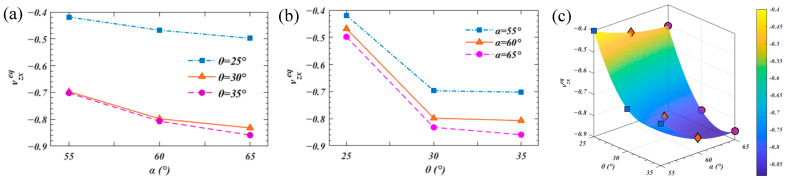
Simulation results of the relationship between angle parameters and the equivalent Poisson’s ratio in the z-direction: (**a**) *α* vs. z-direction equivalent Poisson’s ratio with *θ* fixed; (**b**) *θ* vs. z-direction equivalent Poisson’s ratio with *α* fixed; (**c**) Three-dimensional relationship among *α*, *θ*, and the z-direction equivalent Poisson’s ratio.

Analysis of the static simulation results shows that Figure 11 illustrates the regulatory effect of the angle parameters *α* and *θ* on the equivalent Poisson’s ratio in the z-direction. Within the selected parameter range, the CRNSC structure exhibits negative Poisson’s ratio behavior, with no positive Poisson’s ratio region or characteristic transition inflection point. This is highly consistent with the theoretical calculations, confirming that the CRNSC structure stably exhibits negative Poisson’s ratio characteristics within this parameter range. The single-factor control variable method was employed to investigate the response patterns of the equivalent Poisson’s ratio when *α* and *θ* vary independently. As shown in Figure 11, the simulation results fully reproduce the regulatory patterns revealed by the theoretical calculations within the valid parameter range. The two show strong agreement in variation trends, parameter sensitivity, multi-parameter coupling effects, and numerical magnitudes, fully validating the accuracy and reliability of the theoretical model in predicting the structure’s negative Poisson’s ratio characteristics.

Regarding the regulatory effect of the angle *α*, in three sets of control simulations where *θ* was fixed at 25°, 30°, and 35°, respectively, all curves exhibited the characteristic that as *α* increases, the z-direction equivalent Poisson’s ratio increases strictly and monotonically in the negative direction. The negative Poisson’s ratio characteristic was maintained throughout the entire range, and the rate of change remained stable, consistent with the low-slope, stable variation characteristic in the theoretical calculations for this range. Crucially, within the same range of *α* variation, the larger the *θ* value, the higher the absolute value of the negative equivalent Poisson’s ratio. This characteristic precisely corresponds to the synergistic enhancement mechanism of *α* and *θ* on the negative Poisson’s ratio effect in the theoretical calculations, proving that the theoretical model can accurately capture the variation in the Poisson’s ratio under the coupled action of these two parameters. Furthermore, the characteristic observed in the theoretical calculations—that “an increase in *α* leads to a slower rate of change in the Poisson’s ratio”—is also validated by the simulation results: the absolute change in the three sets of simulation curves within the *α* = 60°~65° range is slightly lower than that in the *α* = 55°~60° range, consistent with the evolution of the rate of change in the theoretical calculations.

Regarding the regulatory effect of angle *θ*, in three sets of control simulations where *α* was fixed at 55°, 60°, and 65°, respectively, within the range of 25°~35° (which coincides with the core regulatory range for the negative Poisson’s ratio in the theoretical calculations), all three sets of curves exhibited a strictly monotonically decreasing pattern: as *θ* increases, the equivalent Poisson’s ratio decreases. The decline remained approximately linear throughout the range, consistent with the linear regulatory characteristics of the theoretical calculations. The core conclusion of the theoretical calculations—that “the sensitivity of *θ* to the Poisson’s ratio is far higher than that of *α*”—is fully reflected in the simulation results. Within the simulation range, a 10° change in the single parameter *θ* resulted in a maximum change in the Poisson’s ratio of 0.46, whereas a 10° change in the single parameter *α* resulted in a maximum change of only 0.15. This difference in regulatory sensitivity between the two parameters is consistent with the theoretical calculation results.

The minor discrepancies between the two primarily stem from three factors. First, the theoretical model adopts the Euler–Bernoulli beam theory to simplify the deformation of the CRNSC structure to the bending and axial deformation of its members, thereby neglecting local shear deformation and stress concentration effects at the nodes. Second, the theoretical model does not account for material nonlinearity, including geometric nonlinearity or plastic deformation, whereas the simulation fully captures the stiffness matrix changes induced by large structural deformations. Third, numerical calculation errors—such as those arising from mesh discretization accuracy and the application of boundary conditions in the simulation—can introduce minor numerical deviations.

### 3.3. Analysis of Effective Young’s Modulus

**Figure 12:**
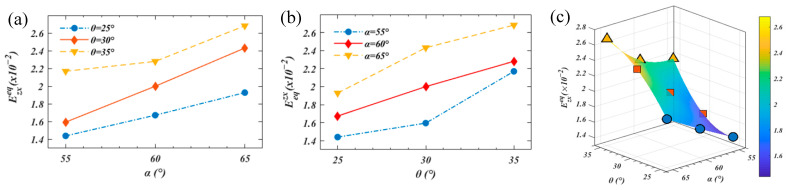
Simulation results showing the relationship between angle parameters and the effective Young’s modulus in the z-direction: (**a**) Relationship between *α* and the effective Young’s modulus in the z-direction when *θ* is fixed; (**b**) Relationship between *θ* and the effective Young’s modulus in the z-direction when *α* is fixed; (**c**) Three-dimensional relationship among *α*, *θ*, and the effective Young’s modulus in the z-direction.

Figure 12 shows the relationship between the angle parameters and the equivalent Poisson’s ratio in the z-direction. Regarding the regulatory effect of angle *θ*, the theoretical calculations and simulation results exhibit consistent variation patterns and parameter sensitivity. In three sets of independent control experiments with *α* fixed at 55°, 60°, and 65°, varying *θ* from 25° to 35° reproduced the monotonically increasing pattern predicted by the theoretical calculations. The simulation results not only matched this monotonic trend but also captured the nonlinear growth characteristics revealed by the theoretical model. Specifically, the absolute increase in modulus within the 30–35° range of *θ* was significantly higher than that in the 25–30° range, consistent with the theoretical prediction that increasing *θ* leads to a continuously rising rate of modulus growth. Furthermore, within the same range of *θ* variation, a higher *α* corresponds to both a higher overall modulus level and a larger absolute increase. This aligns with the synergistic enhancement effect of *α* and *θ* observed in the theoretical calculations, thereby corroborating the accuracy of the theoretical model in predicting the multi-parameter coupling regulation mechanism.

Regarding the regulatory effect of angle *α*, the simulation results align with the single-peak regulatory pattern predicted by the theoretical calculations, validating the theoretical model’s ability to predict non-monotonic variation characteristics. In three sets of independent control experiments with *θ* fixed at 25°, 30°, and 35°, respectively, the variation pattern of α within the 55°~65° range matched the theoretical calculation results. This parameter range precisely covers the pre-peak rising segment and the high-plateau regions around the peak in the theoretical calculations. Consequently, the simulation results for all three control experiments exhibited a monotonically increasing modulus characteristic as *α* increased. The trends in the simulation results align with the pre-peak rising segment of the theoretical calculations for *α* in the 55°~60° range. Meanwhile, the slight increase in modulus observed for *α* in the 60°~65° range does not contradict the single-peak curve pattern of the theoretical calculations. In the theoretical calculations, after *α* reaches its peak at 60°, the rate of modulus decline is extremely slow, and at 65°, the modulus remains above 95% of the peak value, indicating a high-plateau phase. The deviation between the simulation results and the theoretical calculations falls within a reasonable numerical margin and does not violate the single-peak regulation mechanism revealed by the theoretical model. Within the same range of α variation, a higher value of *θ* corresponds to both a higher overall modulus level and a larger absolute increase. This characteristic again verifies the synergistic enhancement effect between *α* and *θ* revealed by the theoretical model, demonstrating that the theoretical model can accurately capture the variation in mechanical properties under the coupled action of the two parameters.

The minor numerical discrepancies between the two primarily stem from two sources. First, during the development of the theoretical model, idealized assumptions were made regarding the structure’s boundary conditions and deformation modes, neglecting the effects of local stress concentrations in the nodal regions and material nonlinear deformation. Second, the finite element simulation fully accounts for large geometric deformations and numerical errors arising from mesh discretization, leading to minor discrepancies with the linear elastic assumptions of the theoretical model. Overall, the numerical discrepancy between the two is well below the 5% error tolerance threshold for engineering applications, demonstrating that the theoretical model possesses excellent quantitative predictive accuracy.

## 4. Static Compression Test of the CRNSC Structure

### 4.1. Materials and Sample Preparation

**Figure 13:**
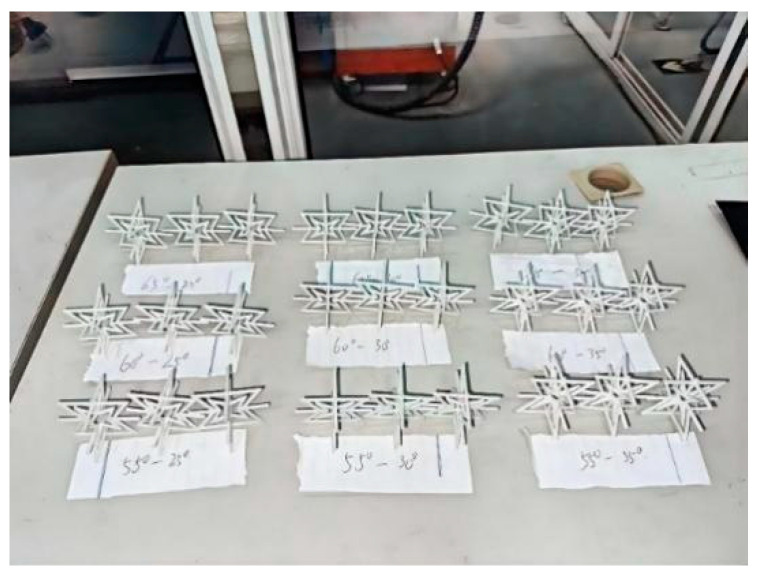
Experimental Specimens.

The test specimens in this experiment were fabricated using stereolithography (SLA) 3D printing technology, with a high-toughness white photopolymer resin serving as the matrix material. The material properties of the specimens are consistent with those used in the finite element simulation described in Section 3. After printing, the specimens were ultrasonically cleaned with anhydrous ethanol to remove residual resin from the surface, followed by post-curing in a UV curing chamber. The key geometric dimensions of the specimens were then measured using a digital vernier caliper, with dimensional deviations controlled within ±0.1 mm. The prepared specimens are shown in Figure 13.

The geometric parameters of the specimens are identical to those of the simulation model in Section 3. An orthogonal experimental design based on angle parameters was adopted, with the angle α (between the outer diagonal members of the unit cell) and the angle *θ* (between the inner diagonal members and the horizontal line) as variables. The remaining geometric parameters were fixed as follows: thickness *δ* = 2 mm, and rod length parameters *L*_3_ = 24 mm, *L*_4_ = 14 mm, and *L*_5_ = 20 mm. To eliminate the influence of 3D printing process variability and dimensional errors on the experimental results, three parallel specimens were prepared for each parameter set, with the specimen numbering convention denoted as (*α* − *θ*).

**Figure 14:**
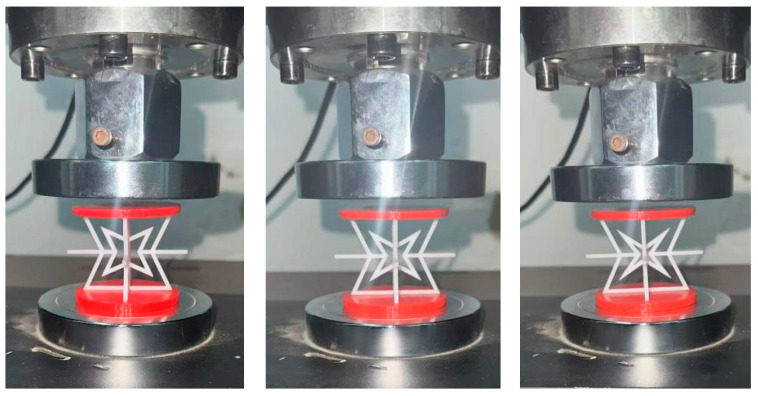
Schematic of CRNSC Solid Compression.

As shown in Figure 14, The quasi-static compression test was conducted on a microprocessor-controlled electronic universal testing machine, model WDW-100D (Jinan Chuanbai Instrument Equipment Co., Ltd., Jinan, China). During the quasi-static loading process, the loading rate was 1 mm/min, and the loading stroke was 50% of the specimen’s initial height. The specimen was placed between the upper and lower jaws, and the upper jaw was adjusted using the fine-tuning wheel until it made full contact with the specimen. Then, the ‘Zero All’ button was clicked, and the test was started.

### 4.2. Stress–Strain Curve Analysis

**Figure 15:**
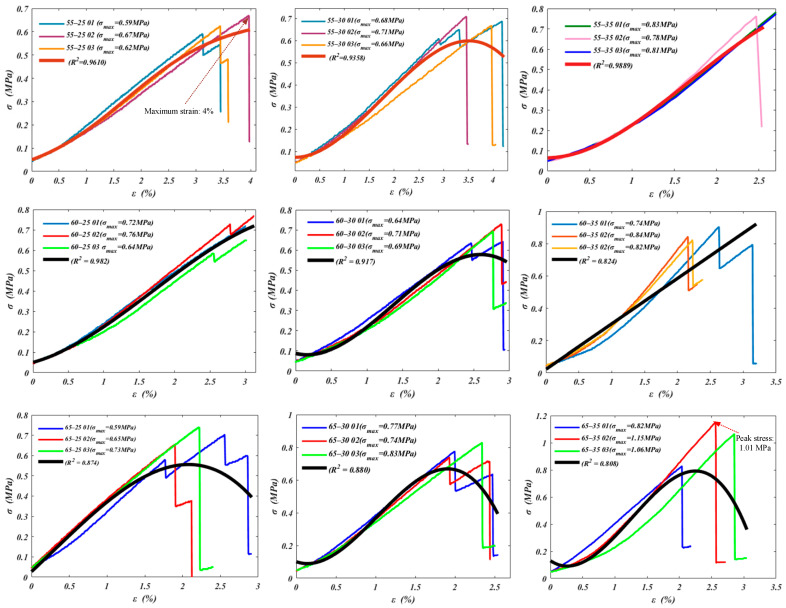
Complete compressive stress–strain curves for 9 parameter groups of specimens.

The complete compressive stress–strain curves for the nine specimen sets are shown in Figure 15. All specimens exhibit typical compressive mechanical behavior of a negative Poisson’s ratio structure, and the compression process can be divided into three stages.

The first stage is the linear elastic stage (strain ≈ 0~2%), where stress and strain show an approximately linear positive correlation. Deformation is mainly caused by rigid-body rotation and slight bending of the re-entrant diagonal members, with no significant plastic deformation or damage. Stress increases rapidly with strain, and the slope remains nearly constant, indicating a stable elastic response.

The second stage is the yield hardening stage (strain > 2%), where the curve slope gradually decreases and the structure enters nonlinear yielding. Bending deformation of the re-entrant diagonal members increases, geometric nonlinear effects become evident, and some specimens show brief stress plateaus or slight hardening. The negative Poisson’s ratio effect peaks in this stage, reflected in the maximum ratio of lateral expansion to axial compression. The angle parameters significantly affect the duration and shape of this stage, highlighting their regulatory role in the nonlinear response.

The third stage is the failure drop stage. After peak stress, member bending failure and joint cracking occur. Stress drops rapidly with increasing strain, and the structure loses its main load-bearing capacity, indicating ultimate failure.

The stress–strain curves of parallel specimens in each group showed good agreement. The coefficient of determination (R^2^) from nonlinear fitting exceeded 0.8 for all groups, reaching 0.989 for the 55–35 group and 0.982 for the 60–25 group, indicating good stability and repeatability of the experimental data. Key mechanical properties, such as peak stress and ultimate strain, are summarized in Table 4.

When *θ* = 25°, the specimen achieves the maximum ultimate strain (4%), showing superior deformation capacity and ductility. When *θ* = 35°, the specimen exhibits the highest overall peak stress; the 65–35 group reaches an average peak stress of 1.01 MPa, indicating the best load-bearing capacity among all groups. This suggests that increasing the internal diagonal angle *θ* significantly enhances structural load-bearing stiffness and peak strength, consistent with theoretical results and simulation analyses.

### 4.3. Analysis of Angular Parameters

**Figure 16:**
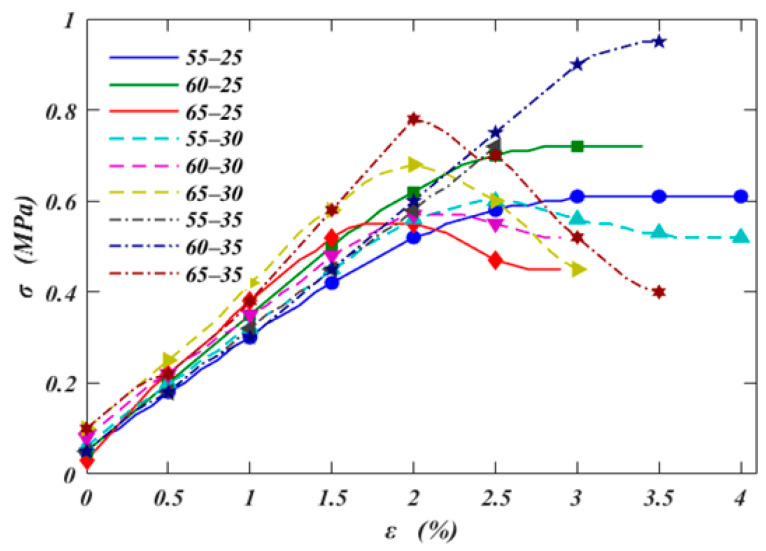
Fitted compressive stress–strain curves for 9 parameter groups of specimens.

The fitted compressive stress–strain curves for the nine specimen sets are shown in Figure 16, matching the original curves well. Key observations are as follows:

Influence of the internal diagonal angle *θ*. Under fixed *α*, the peak stress increases monotonically with *θ*, consistent with simulation results and confirming that *θ* is the key parameter governing load-bearing capacity. Meanwhile, increasing *θ* slightly reduces the ultimate strain. At *θ* = 25°, the ultimate strain reaches 3~4%, whereas at *θ* = 35°, it falls between 2.5% and 3%. This indicates that a larger *θ* enhances load-bearing stiffness but decreases deformation capacity. The reason is that increasing *θ* makes the internal star-shaped structure more compact, reducing the space for member bending deformation and causing earlier failure.

Influence of the external diagonal angle *α*. Under fixed *θ*, the peak stress first increases and then decreases with increasing *α*, peaking at *α* = 60°~65°, which is consistent with simulation results. By adjusting the inclination of the outer load-bearing structure, *α* modifies the force transmission efficiency and stiffness distribution, achieving optimal efficiency within the 60°~65° range and thus maximizing load-bearing capacity. When *α* is too small or too large, the load-bearing path is degraded, leading to a decrease in peak stress. This non-monotonic behavior provides clear parameter guidance for structural optimization design.

### 4.4. Failure Mode Analysis

**Figure 17:**
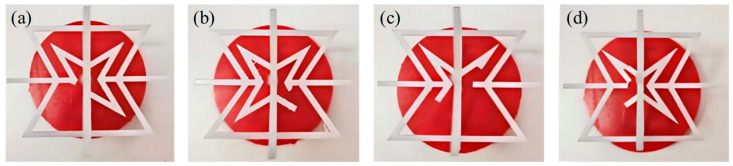
Schematic of Compressive Failure of CRNSC: (**a**) 60–30°; (**b**) 55–30°; (**c**) 60–35°; (**d**) 65–35°.

Figure 17 shows the failure modes of the CRNSC structure during compression testing. Experimental results indicate that failure in all specimens originated at the connections between internal members and nodes. Due to geometric discontinuity, this region constitutes the structural weak point. This finding provides clear direction for subsequent structural optimization. By implementing node fillet optimization, localized thickening, and gradient stiffness distribution, stress concentration in the nodal region can be alleviated, thereby further enhancing the structure’s load-bearing and deformation capacities.

The brittle fracture failure mode primarily occurs in specimens with a high angle (*θ* = 35°), with the 65–35 group being the most typical case. The failure process manifests as sudden brittle cracking at the connection between the member and the joint after the peak stress, accompanied by a rapid load drop, which is classified as a brittle fracture failure mode. Under this failure mode, the high *θ* angle increases the overall stiffness of the structure, constraining the bending deformation of the members and causing stress to concentrate at the joints. When the local stress exceeds the tensile strength of the material, sudden fracture occurs. This failure mode is fully consistent with the simulation analysis results, which identified the joints as stress concentration zones, thereby validating the accuracy of the simulation predictions.

### 4.5. Comparison and Validation of Theoretical Analysis, Simulation, and Experiment

To comprehensively evaluate the accuracy of the theoretical analytical model, the equivalent Poisson’s ratio and the effective Young’s modulus in the z-direction obtained from theoretical calculations, finite element simulations, and experimental measurements were systematically compared. The results are presented in Table 5 and Table 6. Relative error was adopted as the evaluation metric to quantify the degree of agreement among the different methods.

The comparison results show that for the equivalent Poisson’s ratio, the relative error between theoretical calculations and experimental measurements remained within 5%, with a minimum error of 1.25% (65–30 group) and a maximum of 5.00% (55–25 group). Similarly, the relative error between finite element simulations and experimental measurements was also below 5%, ranging from 0.00% (60–25 group) to 4.88% (65–35 group). These results demonstrate that the theoretical model has excellent accuracy in predicting the negative Poisson’s ratio characteristics of the structure and can precisely capture the regulatory effect of the angle parameters on the Poisson’s ratio.

Regarding the effective Young’s modulus, the relative error between theoretical calculations and experimental measurements ranged from 2.13% (65–25 group) to 6.34% (65–35 group). The relative error between finite element simulations and experimental measurements was consistently within 5%, with a maximum of 4.21% (60–30 group). Given the simplifying assumptions of the theoretical model and the complexity of experimental conditions, the maximum error of 6.34% remains within the acceptable range for both academic research and engineering applications (typically less than 10%), fully validating the correctness and predictive capability of the theoretical model.

## 5. Conclusions

This paper proposes a novel Cross Re-entrant Hexagon Structure Nested Star-Shaped Cell (CRNSC). Using a combination of theoretical derivation, finite element simulation, and experimental validation, the influence of the geometric parameters of the CRNSC structure on its equivalent Poisson’s ratio, effective Young’s modulus, and energy absorption characteristics is systematically analyzed. The deformation mechanism and energy absorption mechanism of the structure are elucidated. The main conclusions are as follows:

1. The configuration design and theoretical modeling of the three-dimensional negative Poisson’s ratio CRNSC structure are presented. Analytical expressions for the relative density, the effective Young’s modulus in the x- and z-directions, and the equivalent Poisson’s ratio are derived. Univariate parameter analysis reveals that the thickness parameter *δ* exhibits a significant positive correlation with relative density. The angle parameter *θ* can induce polarity reversal of the Poisson’s ratio and serves as the core sensitive parameter for regulating the negative Poisson’s ratio effect. The angle parameter α exhibits a monotonically negative enhancing effect on the equivalent Poisson’s ratio and a single-peak regulation pattern for the effective Young’s modulus, with the peak occurring in the 55°~60° range.
2. Finite element simulations were conducted using the Ansys Workbench platform. The results indicate that the angle parameter *α* exhibits a monotonically negative enhancing effect on the equivalent Poisson’s ratio in the z-direction and a single-peak regulation pattern for the effective Young’s modulus. The angle parameter *θ* exhibits a strong, linearly monotonically decreasing effect on the equivalent Poisson’s ratio and a nonlinearly increasing effect on the effective Young’s modulus. During compression, the structure exhibits typical negative Poisson’s ratio deformation characteristics, with the deformation mode gradually evolving from rigid-body rotation—which dominates the initial stage—to a coupled mode of bending deformation and rigid-body rotation.
3. Structural specimens were fabricated using stereolithography (SLA) 3D printing technology, and quasi-static uniaxial compression tests were conducted. The experiments indicate that *α* = 65° and *θ* = 35° constitute the optimal load-bearing configuration, with a peak stress of 1.01 MPa, while *α* = 55° and *θ* = 25° constitute the optimal deformation configuration, with a limit strain of 4%. Failure modes are classified into two categories: ductile bending failure and brittle joint fracture failure, both originating at the connections between the members and the joints.

## Figures and Tables

**Table 1:** CRNSC Geometric Parameter Value Range.

Geometric Parameters	*α* (*°*)	*θ* (*°*)	*δ* (mm)	$L_{3}$ (mm)	$L_{4}$ (mm)	$L_{5}$ (mm)
Parameter Range	10–85	10–35	0.5–2	10–30	10–30	10–30

**Table 2:** Selection Range of Geometric Parameters.

*α* (*°*)	*θ* (*°*)	*δ* (mm)	$L_{3}$ (mm)	$L_{4}$ (mm)	$L_{5}$ (mm)
55, 60, 65	25, 30, 35	2	24	14	20

**Table 3:** Material Parameters of High-Toughness ABS White Resin.

Density (kg/m^3^)	Young’s Modulus (GPa)	Poisson’s Ratio	Bulk Modulus (GPa)	Shear Modulus (GPa)	Tensile Yield Strength (MPa)	Compressive Yield Strength (MPa)
1050	1.95	0.41	3.61	0.69	35	38

**Table 4:** Summary of Compressive Mechanical Properties Test Results of Specimens in Each Group.

Sample Number	Peak Stress of the Specimen (MPa)	Peak-to-Peak Stress (MPa)	Ultimate Strain (%)	Curve-Fitting Accuracy R^2^
55–25	0.59, 0.67, 0.62	0.63	~4.0	0.9610
60–25	0.72, 0.76, 0.64	0.71	~3.0	0.9820
65–25	0.59, 0.65, 0.73	0.66	~2.5	0.8740
55–30	0.68, 0.71, 0.66	0.68	~4.0	0.9358
60–30	0.64, 0.71, 0.69	0.68	~3.0	0.9170
65–30	0.77, 0.74, 0.83	0.78	~2.5	0.8800
55–35	0.83, 0.78, 0.81	0.81	~2.5	0.9889
60–35	0.74, 0.84, 0.82	0.80	~3.0	0.8240
65–35	0.82, 1.15, 1.06	1.01	~2.5	0.8080

**Table 5:** Comparison of Equivalent Poisson’s Ratio Results in the z-direction.

Sample Number	Theoretical Calculation	Simulated Value	Experimental Values	Deviation from the Theoretical Value	Error Relative to the Simulation
55–25	−0.42	−0.41	0.40	5.00%	2.50%
60–25	−0.45	−0.47	−0.47	4.26%	0.00%
65–25	−0.48	−0.50	−0.49	2.04%	2.04%
55–30	−0.68	−0.69	−0.67	1.49%	2.99%
60–30	−0.75	−0.76	−0.74	1.35%	2.70%
65–30	−0.81	−0.83	−0.80	1.25%	3.75%
55–35	−0.72	−0.71	−0.70	2.86%	1.43%
60–35	−0.78	−0.80	−0.77	1.30%	3.90%
65–35	−0.84	−0.86	−0.82	2.44%	4.88%

**Table 6:** Comparison of Effective Young’s Modulus Results in the z-direction.

Sample Number	Theoretical Calculation	Simulated Value	Experimental Values	Deviation from the Theoretical Value	Error Relative to the Simulation
55–25	1.48	1.45	1.42	4.23%	2.11%
60–25	1.72	1.70	1.65	4.24%	3.03%
65–25	1.92	1.95	1.88	2.13%	3.72%
55–30	1.63	1.60	1.57	3.82%	1.91%
60–30	1.95	1.98	1.90	2.63%	4.21%
65–30	2.52	2.48	2.40	5.00%	3.33%
55–35	2.23	2.20	2.15	3.72%	2.33%
60–35	2.55	2.52	2.45	4.08%	2.86%
65–35	2.85	2.75	2.68	6.34%	2.61%

## Data Availability

The original contributions presented in this study are included in the article. Further inquiries can be directed to the corresponding author.
